# Supplementary material for: Hidden assumptions of integer ratio analyses in bioacoustics and music
Source: Ann N Y Acad Sci. 2025 Oct 3;1553(1):363–78. doi: 10.1111/nyas.70037 (PMC12645274; doi:10.1111/nyas.70037)
Supplement: Supplementary file 1 — Supplementary material [file NYAS-1553-363-s001.zip › 3_Supporting information S1.pdf]

Hidden assumptions of integer ratio analyses in bioacoustics and music – Supporting Information

Yannick Jadoul[1], Tommaso Tufarelli[2], Chloé Coissac[1], Marco Gamba[3], and Andrea Ravignani[1,4,5]

[1]Department of Human Neurosciences, Sapienza University of Rome, Rome, Italy
[2]Independent researcher
[3]Department of Life Sciences and Systems Biology, University of Turin, Turin, Italy
[4]Center for Music in the Brain, Department of Clinical Medicine, Aarhus University, Aarhus, Denmark
[5]Research Center of Neuroscience "CRiN-Daniel Bovet", Sapienza University of Rome, Rome, Italy
Corresponding author: yannick.jadoul@uniroma1.it

Algorithm S1 Pseudocode to computationally approximate the normalization constant $w_{I,u,v}$. Parameters u and v are the left and right edge of the bin. Parameter n is the number of samples used in the approximation; the higher n the more accurate the estimate.

```
function GetNormalizationConstant(u, v, n)
    m ← 0
    for j = 1, ..., n do
        i1 ← SampleInterval
        i2 ← SampleInterval
        r ← CalculateRhythmRatio(i1, i2)    ▷ Typically, r ← i1/(i1 + i2)
        if u ≤ r ≤ v then
            m ← m + 1
        end if
    end for
    return m/n
end function
```

Code fragment S1 Example Python implementation of the normalization algorithm in Algorithm S1. Concretely, this code calculates the normalization constant of the r_k bin from 0.4 to 0.44... for a uniform distribution of intervals. Adapt variables `u` and `v` to change the bin's edges, and increase `n` to get a more accurate approximation. Change `rng.uniform(...)` to sample from a different null distribution of intervals.

```
import numpy as np

# Boundaries of the left off-ratio bin for 1:1
u, v = 1 / 2.5, 1 / 2.25

# Total number of samples to approximate
n = 10**6

# Create a pseudorandom number generator object
rng = np.random.default_rng()

# Sample from a uniform distribution over [1, 5]
# Change to match the desired null distribution
intervals_1 = rng.uniform(1, 5, size=n)
intervals_2 = rng.uniform(1, 5, size=n)

ratios = intervals_1 / (intervals_1 + intervals_2)
bin_count = np.sum((u <= ratios) & (ratios <= v))
normalization_constant = bin_count / n

print("Normalization constant:", normalization_constant)
```

Code fragment S2 Example R implementation of the normalization algorithm in Algorithm S1. Concretely, this code calculates the normalization constant of the r_k bin from 0.4 to 0.44... for a uniform distribution of intervals. Adapt variables `u` and `v` to change the bin's edges, and increase `n` to get a more accurate approximation. Change `runif(...)` to sample from a different null distribution of intervals.

```
# Boundaries of the left off-ratio bin for 1:1
u <- 1 / 2.5
v <- 1 / 2.25

# Total number of samples to approximate
n = 10^6

# Sample from a uniform distribution over [1, 5]
# Change to match the desired null distribution
intervals_1 <- runif(n, 1, 5)
intervals_2 <- runif(n, 1, 5)

ratios <- intervals_1 / (intervals_1 + intervals_2)
bin_count <- sum((u <= ratios) & (ratios <= v))
normalization_constant <- bin_count / n

paste("Normalization constant:", normalization_constant)
```

Null-hypothesis	Bin (lower)	Bin (upper)	On-/off-integer	Bin count	Normalized bin count	Normalized CI (lower)	Normalized CI (upper)
Poisson process	0.222222	0.235294	Off	159	0.971836	0.770104	1.167487
Poisson process	0.235294	0.266667	On	363	0.924467	0.814944	1.041642
Poisson process	0.266667	0.285714	Off	245	1.027685	0.872462	1.187102
Poisson process	0.285714	0.307692	Off	272	0.988814	0.846981	1.137864
Poisson process	0.307692	0.363636	On	758	1.082554	0.994001	1.175414
Poisson process	0.363636	0.400000	Off	409	0.898650	0.790944	1.004170
Poisson process	0.400000	0.444444	Off	517	0.929410	0.830528	1.021111
Poisson process	0.444444	0.500000	On	1195	1.718600	1.612169	1.823600
Uniform	0.222222	0.235294	Off	159	1.105373	0.875921	1.327908
Uniform	0.235294	0.266667	On	363	0.989779	0.872519	1.115233
Uniform	0.266667	0.285714	Off	245	1.026315	0.871299	1.185519
Uniform	0.285714	0.307692	Off	272	0.931467	0.797860	1.071872
Uniform	0.307692	0.363636	On	758	0.907492	0.833258	0.985335
Uniform	0.363636	0.400000	Off	409	0.652308	0.574127	0.728902
Uniform	0.400000	0.444444	Off	517	0.588671	0.526041	0.646753
Uniform	0.444444	0.500000	On	1195	0.906728	0.850575	0.962125
Log-normal	0.222222	0.235294	Off	159	0.861263	0.682483	1.034653
Log-normal	0.235294	0.266667	On	363	0.793556	0.699543	0.894139
Log-normal	0.266667	0.285714	Off	245	0.856353	0.727009	0.989193
Log-normal	0.285714	0.307692	Off	272	0.808110	0.692197	0.929921
Log-normal	0.307692	0.363636	On	758	0.860421	0.790038	0.934226
Log-normal	0.363636	0.400000	Off	409	0.697910	0.614264	0.779859
Log-normal	0.400000	0.444444	Off	517	0.712881	0.637036	0.783218
Log-normal	0.444444	0.500000	On	1195	1.308058	1.227051	1.387975

Table S1: Full results of the thrush nightingale dataset (main text, Figure 4A), presenting the bin count, normalized bin count, and normalized confidence interval (CI) for each combination of bin and null-hypothesis.

Null-hypothesis	Bin (lower)	Bin (upper)	On-/off-integer	Bin count	Normalized bin count	Normalized CI (lower)	Normalized CI (upper)
Poisson process	0.222222	0.235294	Off	2998	0.428371	0.407936	0.448092
Poisson process	0.235294	0.266667	On	8015	0.477178	0.463305	0.490638
Poisson process	0.266667	0.285714	Off	4814	0.472054	0.454403	0.490592
Poisson process	0.285714	0.307692	Off	10975	0.932701	0.910945	0.956157
Poisson process	0.307692	0.363636	On	38938	1.300008	1.283714	1.313799
Poisson process	0.363636	0.400000	Off	34621	1.778275	1.755516	1.801396
Poisson process	0.400000	0.444444	Off	63944	2.687255	2.664308	2.711337
Poisson process	0.444444	0.500000	On	97432	3.275674	3.254020	3.296454
Uniform	0.222222	0.235294	Off	2998	0.472742	0.450190	0.494505
Uniform	0.235294	0.266667	On	8015	0.475841	0.462006	0.489263
Uniform	0.266667	0.285714	Off	4814	0.424731	0.408850	0.441411
Uniform	0.285714	0.307692	Off	10975	0.776241	0.758134	0.795762
Uniform	0.307692	0.363636	On	38938	0.938307	0.926546	0.948261
Uniform	0.363636	0.400000	Off	34621	1.089860	1.075912	1.104031
Uniform	0.400000	0.444444	Off	63944	1.421378	1.409241	1.434116
Uniform	0.444444	0.500000	On	97432	1.430219	1.420765	1.439292
Log-normal	0.222222	0.235294	Off	2998	1.054677	1.004366	1.103232
Log-normal	0.235294	0.266667	On	8015	0.807426	0.783951	0.830201
Log-normal	0.266667	0.285714	Off	4814	0.564152	0.543058	0.586306
Log-normal	0.285714	0.307692	Off	10975	0.876676	0.856226	0.898723
Log-normal	0.307692	0.363636	On	38938	0.849461	0.838813	0.858472
Log-normal	0.363636	0.400000	Off	34621	0.857888	0.846909	0.869042
Log-normal	0.400000	0.444444	Off	63944	1.091312	1.081993	1.101092
Log-normal	0.444444	0.500000	On	97432	1.192733	1.184849	1.200300

Table S2: Full results of the zebra finch dataset (main text, Figure 4B), presenting the bin count, normalized bin count, and normalized confidence interval (CI) for each combination of bin and null-hypothesis.

Null-hypothesis	Bin (lower)	Bin (upper)	On-/off-integer	Bin count	Normalized bin count	Normalized CI (lower)	Normalized CI (upper)
Poisson process	0.222222	0.235294	Off	340	0.564037	0.477772	0.643666
Poisson process	0.235294	0.266667	On	1205	0.832922	0.775547	0.893767
Poisson process	0.266667	0.285714	Off	658	0.749122	0.678536	0.827683
Poisson process	0.285714	0.307692	Off	829	0.817962	0.749876	0.887035
Poisson process	0.307692	0.363636	On	5394	2.090856	2.027654	2.152491
Poisson process	0.363636	0.400000	Off	3437	2.049649	1.969112	2.128385
Poisson process	0.400000	0.444444	Off	2779	1.355933	1.292503	1.417419
Poisson process	0.444444	0.500000	On	7127	2.781932	2.714792	2.851806
Uniform	0.222222	0.235294	Off	340	0.628959	0.532765	0.717753
Uniform	0.235294	0.266667	On	1205	0.872044	0.811975	0.935747
Uniform	0.266667	0.285714	Off	658	0.730003	0.661219	0.806559
Uniform	0.285714	0.307692	Off	829	0.750884	0.688381	0.814292
Uniform	0.307692	0.363636	On	5394	1.705079	1.653538	1.755341
Uniform	0.363636	0.400000	Off	3437	1.445373	1.388580	1.500896
Uniform	0.400000	0.444444	Off	2779	0.833694	0.794694	0.871498
Uniform	0.444444	0.500000	On	7127	1.423875	1.389511	1.459639
Log-normal	0.222222	0.235294	Off	340	0.666431	0.564506	0.760515
Log-normal	0.235294	0.266667	On	1205	0.827581	0.770574	0.888035
Log-normal	0.266667	0.285714	Off	658	0.632040	0.572486	0.698322
Log-normal	0.285714	0.307692	Off	829	0.617420	0.566027	0.669559
Log-normal	0.307692	0.363636	On	5394	1.336372	1.295976	1.375766
Log-normal	0.363636	0.400000	Off	3437	1.137538	1.092841	1.181236
Log-normal	0.400000	0.444444	Off	2779	0.695322	0.662796	0.726852
Log-normal	0.444444	0.500000	On	7127	1.356875	1.324127	1.390955

Table S3: Full results of the Cuban salsa dataset (main text, Figure 5A), presenting the bin count, normalized bin count, and normalized confidence interval (CI) for each combination of bin and null-hypothesis.

Null-hypothesis	Bin (lower)	Bin (upper)	On-/off-integer	Bin count	Normalized bin count	Normalized CI (lower)	Normalized CI (upper)
Poisson process	0.222222	0.235294	Off	332	0.678619	0.590705	0.774699
Poisson process	0.235294	0.266667	On	459	0.390921	0.346630	0.438620
Poisson process	0.266667	0.285714	Off	393	0.551288	0.486761	0.618628
Poisson process	0.285714	0.307692	Off	630	0.765911	0.685667	0.852234
Poisson process	0.307692	0.363636	On	2649	1.265187	1.206914	1.321074
Poisson process	0.363636	0.400000	Off	2983	2.191859	2.102924	2.288123
Poisson process	0.400000	0.444444	Off	3942	2.369877	2.280300	2.447433
Poisson process	0.444444	0.500000	On	6016	2.893390	2.818843	2.972756
Uniform	0.222222	0.235294	Off	332	0.741848	0.645743	0.846879
Uniform	0.235294	0.266667	On	459	0.389394	0.345275	0.436906
Uniform	0.266667	0.285714	Off	393	0.498755	0.440377	0.559678
Uniform	0.285714	0.307692	Off	630	0.643388	0.575980	0.715902
Uniform	0.307692	0.363636	On	2649	0.926259	0.883596	0.967174
Uniform	0.363636	0.400000	Off	2983	1.367593	1.312103	1.427656
Uniform	0.400000	0.444444	Off	3942	1.278743	1.230409	1.320591
Uniform	0.444444	0.500000	On	6016	1.290888	1.257629	1.326297
Log-normal	0.222222	0.235294	Off	332	1.062150	0.924550	1.212530
Log-normal	0.235294	0.266667	On	459	0.473538	0.419886	0.531317
Log-normal	0.266667	0.285714	Off	393	0.525177	0.463706	0.589328
Log-normal	0.285714	0.307692	Off	630	0.618870	0.554031	0.688621
Log-normal	0.307692	0.363636	On	2649	0.798110	0.761350	0.833365
Log-normal	0.363636	0.400000	Off	2983	1.122131	1.076600	1.171413
Log-normal	0.400000	0.444444	Off	3942	1.078591	1.037822	1.113889
Log-normal	0.444444	0.500000	On	6016	1.222216	1.190726	1.255742

Table S4: Full results of the Malian jembe dataset (main text, Figure 5B), presenting the bin count, normalized bin count, and normalized confidence interval (CI) for each combination of bin and null-hypothesis.

S1 Full derivation of probability distributions for uniformly distributed intervals

Assume intervals are distributed uniformly over $[a, b]$. I.e., the probability density of intervals p_I is:

$$p_I(i) = \begin{cases} \frac{1}{b-a} & \text{if } a \leq i \leq b \\ 0 & \text{otherwise} \end{cases} \tag{1}$$

To determine the distribution p_Q of $q = \frac{i_2}{i_1}$, we calculate the following integral:

$$p_Q(q) = \int_0^{\infty} t\, p_I(t)\, p_I(q\,t)\, dt \tag{2}$$

If $0 < q < \frac{a}{b}$ or $q > \frac{b}{a}$, there is no way to have both $a \leq t \leq b$ and $a \leq q\,t \leq b$.

- If $0 < q < \frac{a}{b}$:

$$a \leq q\,t < \frac{a}{b}t \Rightarrow t > \frac{b}{a}a = b$$
$$t \leq b \Rightarrow q\,t \leq q\,b < \frac{a}{b}b = a$$

- If $q > \frac{b}{a}$:

$$b \geq q\,t > \frac{b}{a}t \Rightarrow t < \frac{a}{b}b = a$$
$$t \geq a \Rightarrow q\,t \geq q\,a > \frac{b}{a}a = b$$

So, for $0 < q < \frac{a}{b}$ or $q > \frac{b}{a}$, either $p_I(t) = 0$ or $p_I(q\,t) = 0$ and consequently $p_Q(q) = 0$. So we just need to calculate $p_Q(q)$ for $\frac{a}{b} \leq q \leq \frac{b}{a}$. There are again two cases:

- If $\frac{a}{b} \leq q \leq 1$:

$$p_I(t) > 0 \Leftrightarrow a \leq t \leq b$$
$$p_I(q\,t) > 0 \Leftrightarrow \frac{a}{q} \leq t \leq \frac{b}{q}$$
$$p_I(t)\, p_I(q\,t) > 0 \Leftrightarrow \frac{a}{q} \leq t \leq b$$

So, we can reduce the interval of integration to $[\frac{a}{q}, b]$:

$$\begin{aligned} p_Q(q) &= \int_{\frac{a}{q}}^{b} t \left(\frac{1}{b-a}\right)\left(\frac{1}{b-a}\right) dt \\ &= \frac{1}{(b-a)^2} \int_{\frac{a}{q}}^{b} t\,dt \\ &= \frac{1}{2(b-a)^2}\left[b^2 - \left(\frac{a}{q}\right)^2\right] \end{aligned}$$

- Symmetrically, if $1 \le q \le \frac{b}{a}$:

$$p_I(t)\, p_I(q\,t) > 0 \Leftrightarrow a \le t \le \frac{b}{q}$$

and

$$\begin{aligned} p_Q(q) &= \int_{a}^{\frac{b}{q}} t \left(\frac{1}{b-a}\right)\left(\frac{1}{b-a}\right) dt \\ &= \frac{1}{(b-a)^2} \int_{a}^{\frac{b}{q}} t\,dt \\ &= \frac{1}{2(b-a)^2}\left[\left(\frac{b}{q}\right)^2 - a^2\right] \end{aligned}$$

Altogether, $p_Q(q)$ becomes

$$p_Q(q) = \begin{cases} \frac{1}{2(b-a)^2}\left[b^2 - \left(\frac{a}{q}\right)^2\right] & \text{if } \frac{a}{b} \le q < 1 \\ \frac{1}{2(b-a)^2}\left[\left(\frac{b}{q}\right)^2 - a^2\right] & \text{if } 1 \le q \le \frac{b}{a} \\ 0 & \text{otherwise} \end{cases} \quad (3)$$

The cumulative probability distribution of q is

$$P_Q(q) = \int_0^q p_Q(t)\,dt$$
$$= \begin{cases} 0 & \text{if } 0 \le q < \frac{a}{b} \\ \int_{\frac{a}{b}}^{q} p_Q(t)\,dt & \text{if } \frac{a}{b} \le q < 1 \\ 1 - \int_q^{\frac{b}{a}} p_Q(t)\,dt & \text{if } 1 \le q < \frac{b}{a} \\ 1 & \text{if } \frac{b}{a} \le q \end{cases}$$
$$= \begin{cases} 0 & \text{if } 0 \le q < \frac{a}{b} \\ \frac{1}{2(b-a)^2}\left[qb^2 + \frac{a^2}{q} - 2ab\right] & \text{if } \frac{a}{b} \le q < 1 \\ 1 - \frac{1}{2(b-a)^2}\left[\frac{b^2}{q} + qa^2 - 2ab\right] & \text{if } 1 \le q < \frac{b}{a} \\ 1 & \text{if } \frac{b}{a} \le q \end{cases} \tag{4}$$

This formulation of $P_Q(q)$ then determines the two rescaling formulas $f_+(q)$ and $f_-(q)$, that map a uniform distribution of intervals into uniform distribution of ratios s:

$$f_+(q) = P_Q(q) \tag{5}$$
$$f_-(q) = 1 - P_Q(q) \tag{6}$$

Performing the change of variable according to ratio transformation $r = f(q) = \frac{1}{1+q}$ or $q = f^{-1}(r) = \frac{1}{r} - 1$, we get probability distribution $p_R(r)$, as plotted in the main manuscript's figures:

$$p_R(r) = p_Q\left(\frac{1}{r} - 1\right)\left|\frac{d}{dr}\left(\frac{1}{r} - 1\right)\right|$$
$$= \frac{1}{r^2}\,p_Q\left(\frac{1-r}{r}\right)$$
$$= \begin{cases} \frac{1}{2(b-a)^2}\left[\left(\frac{b}{1-r}\right)^2 - \left(\frac{a}{r}\right)^2\right] & \text{if } \frac{a}{a+b} \le r < \frac{1}{2} \\ \frac{1}{2(b-a)^2}\left[\left(\frac{b}{r}\right)^2 - \left(\frac{a}{1-r}\right)^2\right] & \text{if } \frac{1}{2} \le r \le \frac{b}{a+b} \\ 0 & \text{otherwise} \end{cases} \tag{7}$$

To get the total probability in a bin $[u, v]$, we integrate $p_R(r)$, assuming $\frac{a}{a+b} \le u \le v \le \frac{b}{a+b}$:

$$\int_u^v p_R(r) = \begin{cases} \frac{1}{2(b-a)^2}\left[\frac{b^2}{1-v} + \frac{a^2}{v} - \frac{b^2}{1-u} - \frac{a^2}{u}\right] & \text{if } \frac{a}{a+b} \le u \le v < \frac{1}{2} \\ \frac{1}{2(b-a)^2}\left[-\frac{b^2}{v} - \frac{a^2}{1-v} + \frac{b^2}{u} + \frac{a^2}{1-u}\right] & \text{if } \frac{1}{2} \le u \le v \le \frac{b}{a+b} \\ \frac{1}{2(b-a)^2}\left[4b^2 + 4a^2 - \frac{b^2}{1-u} - \frac{a^2}{u} - \frac{b^2}{v} - \frac{a^2}{1-v}\right] & \text{if } \frac{a}{a+b} \le u \le \frac{1}{2} \le v \le \frac{b}{a+b} \end{cases} \tag{8}$$

An alternative, more elegant way to describe $\int_u^v p_R(r)\,dr$ is to calculate the cumulative probability distribution $P_R(r)$:

$$\begin{aligned}
P_R(r) &= \int_0^r p_R(t)\,dt \\
&= \begin{cases} 0 & \text{if } 0 \leq r < \frac{a}{a+b} \\ \int_{\frac{a}{a+b}}^{r} p_R(t)\,dt & \text{if } \frac{a}{a+b} \leq r < \frac{1}{2} \\ 1 - \int_{\frac{1}{2}}^{\frac{b}{a+b}} p_R(t)\,dt & \text{if } \frac{1}{2} \leq r < \frac{b}{a+b} \\ 1 & \text{if } \frac{a}{a+b} \leq r \leq 1 \end{cases} \\
&= \begin{cases} 0 & \text{if } 0 \leq r < \frac{a}{a+b} \\ \frac{1}{2(b-a)^2}\left[\frac{b^2}{1-r} + \frac{a^2}{r} - (a+b)^2\right] & \text{if } \frac{a}{a+b} \leq r < \frac{1}{2} \\ 1 - \frac{1}{2(b-a)^2}\left[\frac{b^2}{r} + \frac{a^2}{1-r} - (a+b)^2\right] & \text{if } \frac{1}{2} \leq r < \frac{b}{a+b} \\ 1 & \text{if } \frac{a}{a+b} \leq r \leq 1 \end{cases}
\end{aligned} \tag{9}$$

Now, the area under the curve in interval $[u, v]$ is equal to $P_R(v) - P_R(u)$.

For example, in the case of the bins used by Roeske et al. (2020) and others, these are the normalization constants $\hat{w}_{I,u,v}$ for, respectively, the on-ratio and off-ratio bins around the 1:1 ratio:

$$\hat{w}_{I,0.44\ldots,0.5} = \hat{w}_{I,0.5,0.55\ldots} = \frac{1}{2} - \frac{(4b-5a)^2}{40(b-a)^2} \tag{10}$$

$$\hat{w}_{I,0.4,0.44\ldots} = \hat{w}_{I,0.55\ldots,0.6} = \frac{(4b-5a)^2}{40(b-a)^2} - \frac{(2b-3a)^2}{12(b-a)^2} \tag{11}$$

References

Roeske, T. C., Tchernichovski, O., Poeppel, D., & Jacoby, N. (2020). Categorical rhythms are shared between songbirds and humans. *Current Biology*, *30*(18), 3544–3555.e6. https://doi.org/10.1016/j.cub.2020.06.072
